# Immune Checkpoint-Induced Colitis: A Single-Center Retrospective Cohort Study

**DOI:** 10.3390/jcm14207219

**Published:** 2025-10-13

**Authors:** Bengt Van Holder, Julie Vereecke, Nathan Ureel, Triana Lobatón, Anne Hoorens, Amber Lievens, Marie Truyens, Sylvie Rottey, Karim Vermaelen, Venita D’Cruz, Jeroen Geldof, Celine Jacobs, Eline Naert, Elien De Mulder, Michael Saerens

**Affiliations:** 1 Department of Medical Oncology, Ghent University Hospital, 9000 Ghent, Belgium; sylvie.rottey@uzgent.be (S.R.); celine.jacobs@uzgent.be (C.J.); eline.naert@uzgent.be (E.N.); elien.demulder@uzgent.be (E.D.M.); 2 Faculty of Medicine and Health Sciences, Ghent University, 9000 Ghent, Belgium; nathan.ureel@ugent.be (N.U.); triana.lobatonortega@uzgent.be (T.L.); anne.hoorens@uzgent.be (A.H.); amber.lievens@uzgent.be (A.L.); marie.truyens@uzgent.be (M.T.); karim.vermaelen@uzgent.be (K.V.); jeroen.geldof@uzgent.be (J.G.); 3 Department of Gastroenterology and Hepatology, Ghent University Hospital, 9000 Ghent, Belgium; 4 Department of Pathology, Ghent University Hospital, 9000 Ghent, Belgium; 5 Respiratory Medicine, Ghent University Hospital, 9000 Ghent, Belgium

**Keywords:** immunotherapy, immune-related adverse events, colitis, infliximab, vedolizumab

## Abstract

**Background**: Immune checkpoint inhibitors (ICIs) have transformed cancer therapy but are often complicated by immune-related adverse events, particularly colitis. With increasing ICI use, understanding the clinical course and management of ICI-induced colitis is essential. **Objectives**: To characterize the clinical, endoscopic, and histological features of ICI-induced colitis and evaluate treatment outcomes, focusing on the use of corticosteroids and second-line biologicals (infliximab and vedolizumab) in a real-world setting. **Methods**: A retrospective cohort study was conducted at Ghent University Hospital, including 77 adult patients diagnosed with ICI-induced colitis in between 2012 and 2023. Clinical, biochemical, endoscopic, and histological data were analyzed, along with treatment response and safety outcomes. **Results**: Patients with ICI-induced colitis received anti-PD-1/PD-L1 (64.9%), anti-CTLA-4 (9.1%), or combination of both (26.0%). In patients with normal endoscopic findings, histological signs of colitis were observed in 88.0%. Combination ICI therapy was associated with higher Mayo scores (*p* = 0.029) and increased need for biologicals (*p* = 0.011) compared to anti-PD-1/PD-L1 monotherapy. Clinical response rates were 79.6% with corticosteroids and 100.0% with biologicals. Rechallenge with ICIs lead to a 17.4% relapse rate. No colitis-related deaths were observed. **Conclusions**: In this retrospective study, we demonstrate that random colon biopsies reveal microscopic ICI-induced colitis in most patients with absence of endoscopic disease. Combination ICI therapy predicts a corticosteroid-refractory course, supporting the need for early escalation to biologicals. ICI rechallenge appears feasible, as relapse rates were relatively low and colitis morbidity remained manageable. Prospective studies are needed to refine therapeutic strategies and improve patient outcomes.

## 1. Introduction

Immune checkpoint inhibitors (ICIs) revolutionized the treatment of many cancer types. By targeting intrinsic down-regulators of immunity, such as cytotoxic T-lymphocyte antigen 4 (CTLA-4), lymphocyte activating gene 3 (LAG-3), and programmed cell death 1 (PD-1) or its ligand PD-L1, ICIs enhance antitumor immunity by disrupting immunosuppressive signaling pathways. Inhibiting these self-protective regulators predisposes the patient to a specific type of adverse events inherent to ICIs, namely immune-related adverse events (irAEs) [1,2].

Among irAEs, gastro-intestinal (GI) toxicities are one of the most common, and tend to occur more frequently with anti-CTLA-4-based treatment [1,3]. Symptoms of GI irAEs may be abdominal pain, bloating, vomitus, diarrhea, slimy stools, or gross GI blood loss. Any part of the GI tract may be involved, yet the most frequent form is colitis [4].

ICI-induced colitis occurs at a median of 7 weeks after treatment initiation, but delayed manifestation even after discontinuation of ICIs has been documented [5]. Severe complications, such as perforations, life-threatening electrolyte disturbances, or hypovolemic shock, may occur and may be fatal for patients with ICI-induced colitis [6]. The differential diagnosis includes GI infections and other causes of treatment-related diarrhea, such as chemotherapy, tyrosine kinase inhibitors, and radiotherapy. A stool analysis for bacterial enteropathogens and clostridioides difficile toxin should be conducted in all patients receiving ICIs who present with diarrhea. Disease severity should be graded using the Common Terminology Criteria for Adverse Events (CTCAE) v5.01 scale (Table S1, see Supplementary Materials). A flexible endoscopy with biopsies is mandatory to confirm the diagnosis and establish histopathologic characterization [7].

ICI-induced colitis is a histopathological heterogeneous disease with different microscopic findings. These findings can be classified into different subtypes (Table 1). However, difficulties in diagnostic workout can occur as these histological features overlap with other inflammatory conditions [8].

Management strategies vary according to severity and are outlined in multiple guidelines [7,9,10,11]. In general, withholding ICIs and corticosteroids administration form the mainstay of treatment, alongside supportive care (fluids, electrolytes, and dietary modifications). In case of inadequate response, treatment with biologicals is necessary. Infliximab, a monoclonal antibody targeting tumor necrosis factor alpha (TNFα), is usually considered as the preferred second-line therapy, due to its ability to induce a rapid clinical response, analogous to its established efficacy in the treatment of inflammatory bowel disease (IBD) [12,13]. However, TNFα blockers may cause severe immunosuppression, exposing the patient to potential opportunistic infections, especially if combined with protracted steroid courses [14].

Vedolizumab is a monoclonal antibody directed towards the α4β7 integrin and is an appealing alternative strategy for infliximab with the advantage of gut-specific immunosuppression, while the immunity in extraintestinal tissues remains intact [15]. The efficacy of vedolizumab in treating ICI-induced colitis has been documented, but the evidence is less ubiquitous [16,17,18]. A retrospective study of 184 patients with ICI-induced colitis reported comparable colitis remission rates and an improved overall survival for vedolizumab, compared to infliximab [14].

As the number of patients receiving ICIs continues to rise, it is increasingly important to understand the burden of ICI-induced colitis. Prospective studies remain scarce, and most available evidence comes from retrospective cohorts. Few studies have provided a comprehensive assessment that integrates the clinical, endoscopic, and histological findings, nor have they systematically examined how these parameters correlate with treatment outcomes. Moreover, there is an ongoing need for real-world data to clarify the clinical course and long-term outcomes of patients with irAEs.

In this retrospective cohort study, we evaluated patients treated for ICI-induced colitis in a tertiary care facility in Ghent, Belgium. The objectives were to characterize the clinical, endoscopic, and histological pattern of ICI-induced colitis and to correlate these to the treatment response. A special focus was given to those requiring an escalation beyond corticosteroids with secondary immunosuppressants. We also described safety outcomes including complications related to the ICI colitis and oncological outcome.

In addition to these descriptive analyses, we focused on three key clinically relevant questions for which current evidence is limited, as follows:

1. Diagnostic value of biopsies when endoscopic findings are normal: Among patients with endoscopically normal mucosa, how often is histological inflammation present? This is an important gap in current knowledge, as normal endoscopic findings may not reliably exclude ICI-induced colitis and could potentially misguide treatment decisions.
2. Impact of ICI regimen: Is combination therapy with anti-CTLA-4 and anti-PD-1/PD-L1 associated with a more steroid-refractory disease course compared with anti-PD-1/PD-L1 monotherapy? This is clinically relevant because the type of ICI regimen may predict the need for escalation to treatment with biologicals.
3. Risk of relapse upon rechallenge: What proportion of patients experience recurrent colitis after ICI rechallenge? Although data are limited, this information is crucial for shared decision-making when considering retreatment with ICIs.

## 2. Materials and Methods

### 2.1. Study Design and Setting

The study design is a single-center retrospective cohort study.

### 2.2. Participants

Adult patients (≥18 years) who were treated in the Ghent University Hospital with an ICI from 2012 to 2023 and presented with ≥grade 1 diarrhea compatible with ICI-induced colitis and had undergone a lower gastro-intestinal endoscopy (sigmoidoscopy or ileocolonoscopy) were eligible for inclusion. We defined colitis as ICI-induced in patients who had at least one treatment with an ICI before the onset of colitis with clinical (abdominal pain, bloating, vomitus, diarrhea, and bloody/slimy stools) and/or endoscopic (erythema, erosions, ulcerations, friability, and loss of vascularization pattern) and/or histological signs of colonic inflammation with exclusion of GI infection (negative coproculture and clostridioides difficile toxin). Patients were classified according to treatment group (anti-PD-1/PD-L1 monotherapy, anti-CTLA-4 monotherapy, or anti-PD-1/PD-L1 + anti-CTLA-4 combination therapy). Patients who received combination treatments with chemotherapy, targeted therapy, or an investigational agent were allocated to the cohort that matched their ICI regimen. Patients with multiple irAEs were eligible for inclusion.

### 2.3. Data Collection

Histopathologic specimens were retrieved from the Department of Pathology and were reviewed by an experienced pathologist. To identify relevant cases, a search was conducted within the pathology reports using the following keywords: ‘irAE’, ‘immuun therapie’, ‘immuuntherapie’ and ‘immunotherapie’. Clinical data were manually collected from the electronic health record. Data were extracted by the treating physicians in November 2024. The collected data included baseline characteristics (demographics, clinical characteristics, and tumor characteristics), colitis characteristics, including date of onset, lab values, endoscopic findings, histological pattern (active/infectious-type, chronic/IBD-type, lymphocytic-type, or collagenous-type colitis pattern), and details on the treatment and outcome of the irAE event. Symptom severity was assessed following the CTCAE criteria v.5.0 for diarrhea. The most severe score during the episode of colitis was recorded. Endoscopy was conducted by gastroenterologists with expertise and experience in inflammatory bowel disease and was assessed using the Mayo endoscopic subscore. This score includes 4 grades of endoscopic activity (Table S2). A complete list of the extracted data can be found in the Supplementary Materials.

### 2.4. Outcomes of Interest and Definitions

Main outcomes of interest were the clinical response rate, which is defined as the decrease in symptom severity, and clinical remission rate, defined as a normalization of stool frequency and absence of abdominal symptoms, both according to physician assessment. Other main outcomes include the rate of biochemical response and remission, based on C-reactive protein (CRP) and/or fecal calprotectin and endoscopic response and remission, based on the Mayo score. Response and remission were assessed after initiation of treatment for ICI-induced colitis at times of interest, determined by the treating physician. These outcomes of interest are defined in Table 2.

Other outcomes of interest were the need for biologicals for ICI-induced colitis, responses, safety outcomes (i.e., risk of hospitalization, secondary infections, and treatment-related death), and oncologic outcome (progression-free survival).

Special outcomes of interest were the need for biologicals compared by type of ICI regimen, the proportion of aberrant histological findings in patients with Mayo 0, and the risk of relapse colitis after rechallenge with ICIs.

### 2.5. Data Analysis

Data collection was performed using REDCap^®^ (Research Electronic Data Capture), a web-based application designed for data capture and management in research studies. All the collected data were organized in an encoded SPSS database. Our findings were reported in accordance with the STROBE guidelines [19].

Statistical analysis was conducted using IBM SPSS Version 29.0.2.0^®^ (New York, NY, USA). Descriptive analysis was performed in SPSS to identify frequencies of demographic variables, clinicopathologic variables, and adverse events. For central tendency measures of continuous variables, we used mean for normal distributed variables and median for non-normal distributed variables. Testing for normality was conducted by using the Shapiro–Wilk test, followed by visual confirmation on a Q-Q plot. Distribution of categorical variables was summarized by frequencies and percentages. Continuous variables were compared between subgroups using the Mann–Whitney U test or Kruskal–Wallis test (for more than two groups). Fisher’s Exact test (if expected cell frequency < 5 in at least 80% of cells) or Chi^2^ test (for more than two groups) was used to evaluate associations between categorical variables. Kaplan Meier curves were used to estimate oncological progression-free survival and overall survival. Cox regression analysis was used to calculate hazard ratios. All statistical tests were 2-sided. *p* values < 0.05 were considered statistically significant. An overview of all performed hypothesis tests can be found in the Supplementary Materials.

## 3. Results

### 3.1. Patient Characteristics

**Figure 1:**
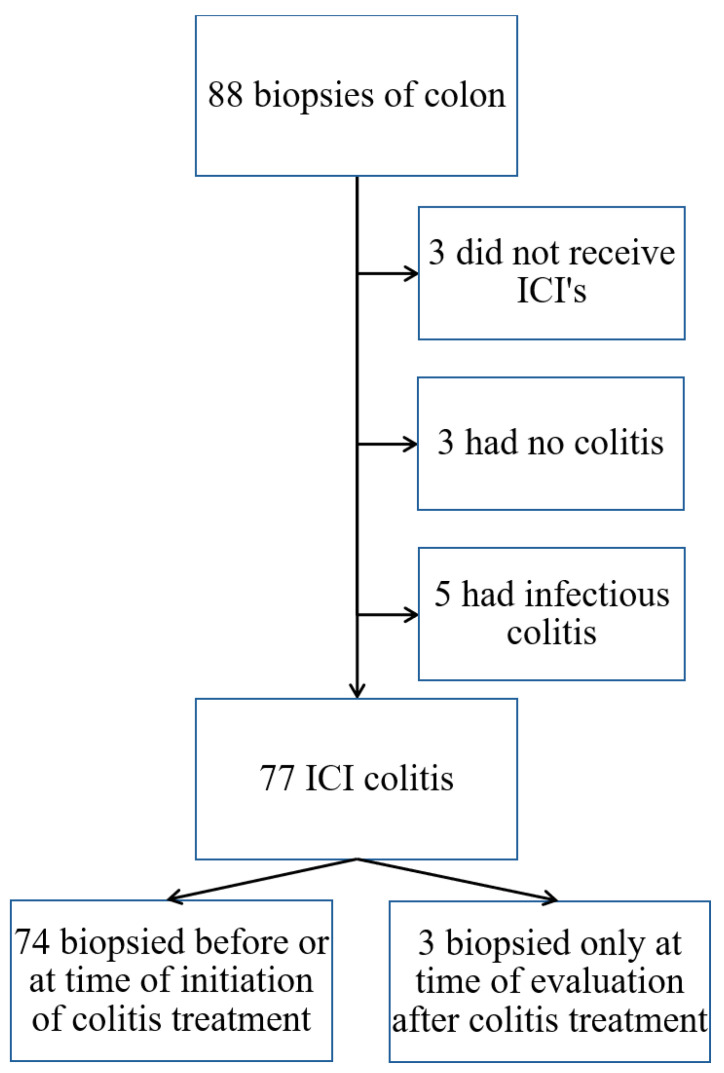
PRISMA flow chart: patients included with ICI-induced colitis.

Of the 88 biopsies identified, 77 patients were included for analysis, of whom three underwent biopsy only at the time of evaluation after treatment for ICI-induced colitis (Figure 1).

The median age was 64 years, with a slight male predominance (57.1%). Melanoma (31.2%), lung cancer (20.8%), and renal cell carcinoma (18.2%) were the most common tumor types. Most patients (87%) received ICIs for advanced disease. Anti-PD-1/PD-L1 monotherapy accounted for 64.9% of cases, while 26% received combination regimens with anti-CTLA-4 (Table 3). Concomitant irAEs were frequent, affecting nearly one third of patients (32.5%) (Table S3).

These baseline data demonstrate the heterogeneity of our study population, with most patients in the metastatic setting and anti-PD-1/PD-L1 monotherapy as the most prevalent regimen.

A comprehensive list of investigational drugs can be found in the Supplementary Materials [20,21,22,23].

### 3.2. Clinical Presentation and Diagnostic Findings

To characterize the clinical features of ICI-induced colitis, we investigated the clinical, endoscopic, biochemical, and histopathological characteristics.

**Figure 2:**
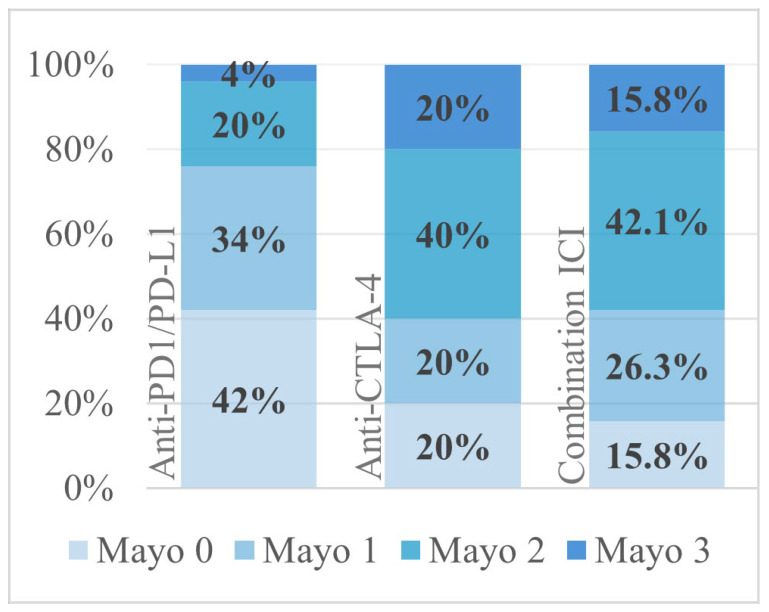
Distribution of the Mayo score for the different ICI regimens.

Colitis developed after a median of 71 days (IQR 29–185 days) from ICI initiation, indicating that most cases arise within the first months, but late presentations remain common and warrant continuous monitoring for symptoms. Most patients presented with grade 2 diarrhea (49.4%). Endoscopy revealed no visible inflammation (Mayo 0) in one-third of patients (33.8%), whereas 36.5% had moderate-to-severe findings (Mayo 2–3). Patients receiving combination therapy had higher Mayo scores compared to monotherapy (*p* = 0.029) although diarrhea grade was not different between treatment groups (*p* = 0.261) (Figure 2).

**Figure 3:**
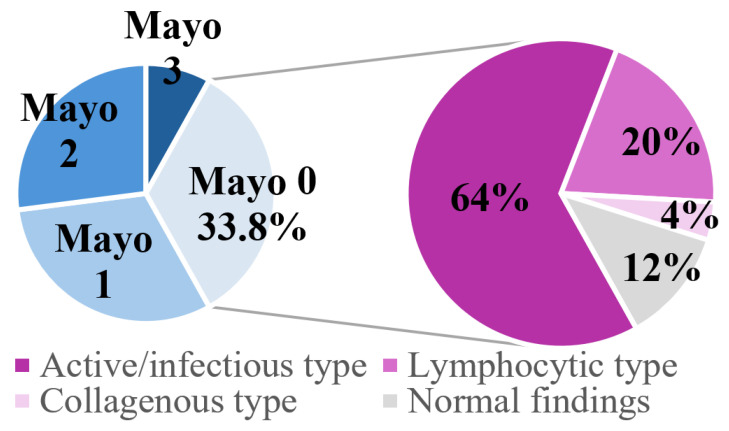
Distribution of the Mayo score at diagnosis of ICI-induced colitis and the histopathologic differentiation in patients with a Mayo score of zero. Mayo 0 in lightest blue; Mayo 1 in light blue, Mayo 2 in dark blue, Mayo 3 in darkest blue.

Histopathology revealed active/infectious-type colitis in 64.9% and apoptotic features in 47.3%. There was no significant association between presence of apoptosis and CRP (*p* = 0.272), fecal calprotectin (*p* = 0.161), grade of diarrhea (*p* = 0.137), Mayo score (*p* = 0.937), and ICI regimen (anti-PD-1/PD-L1 compared to combination anti-PD1/PD-L1 and anti-CTLA-4) (*p* = 0.789), indicating that apoptosis is a unique diagnostic finding that is not captured by clinical, endoscopic, or biochemical assessments. Strikingly, 88% of patients with Mayo 0 had histopathological signs of inflammation or apoptosis, underscoring that normal endoscopic appearance does not exclude microscopic disease (Figure 3).

Upon diagnosis, an elevated CRP level (>5 mg/L) was observed in 80% of patients, while 68% showed increased calprotectin levels (>250 µg/g) at diagnosis, with median levels of 28.9 mg/L and 323 µg/g, respectively (Table 3). Chronic/IBD-type colitis pattern is associated with a higher fecal calprotectin at time of diagnosis compared to the other histopathological patterns (*p* = 0.002) (Figure S1). No significant differences were found for CRP between the different histological patterns (*p* = 0.295) (Figure S2).

### 3.3. Treatment of ICI-Induced Colitis

#### 3.3.1. First-Line Treatment

To characterize the outcome of ICI-induced colitis, we assessed the clinical, biochemical, and histological response after treatment (see Table 2 for definitions).

#### 3.3.2. Corticosteroids

**Figure 4:**
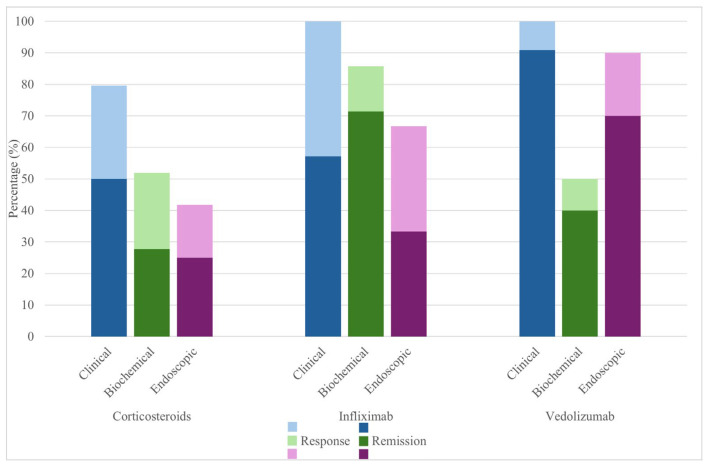
Clinical, biochemical, and endoscopic response/remission for patients treated with corticosteroids and/or biologicals.

Systemic corticosteroids were initiated in 59/77 (76.6%) of patients, and another 7 (9%) patients were treated with local-acting corticosteroids only. The majority received systemic steroids in monotherapy, and some received additional local or systemic immunosuppressants (Table 4). Steroids were initiated after a median of 9 days (IQR 5–20.25 days). The median duration of steroid treatment was 70 days (IQR 47–132 days) and the median starting dose was 1.27 mg/kg prednisolone or equivalent (IQR 0.74–1.84 mg/kg). Among the 59 patients treated with corticosteroids for ICI-induced colitis, only 54 underwent an assessment of treatment response. Clinical response occurred in 79.6%, with a median onset of 13 days. Biochemical and endoscopic responses were more modest (51.9% and 41.7%, respectively), and remission rates were lower (27.8% biochemical, and 25% endoscopic) (Figure 4 and Table 5 and Table 6). Of note, the majority of corticosteroid-only treated patients had no repeated endoscopy whilst having a clinical response, suggesting an underestimation of endoscopic response and remission rates.

#### 3.3.3. Second-Line Biologicals for Steroid-Refractory Disease

We next examined outcomes with infliximab and vedolizumab in patients failing corticosteroids.

**Figure 5:**
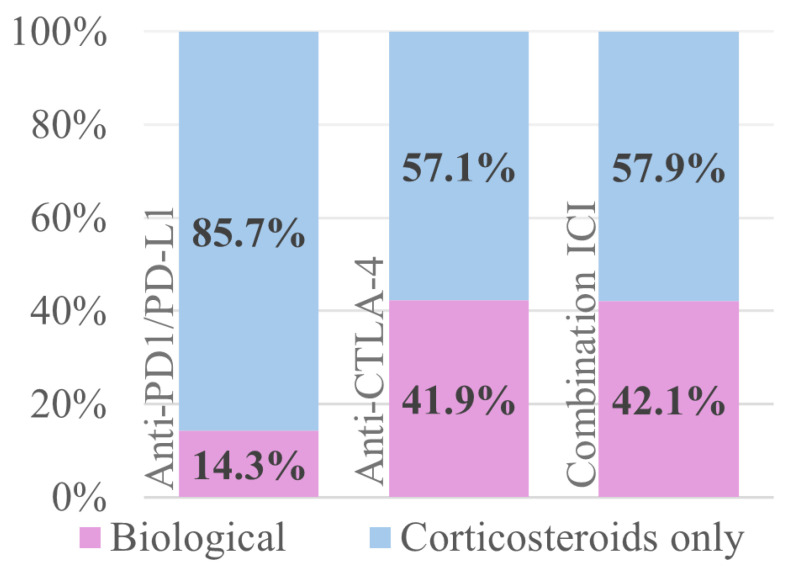
Distribution of the need for biologicals for the different ICI regimens.

In total, 19/77 (24.7%) patients needed biologicals as additional treatment, either infliximab (*n* = 7), vedolizumab (*n* = 11), or both sequentially (*n* = 1). Infliximab induced rapid responses (median 7 days), with 100% clinical improvement and 57.1% clinical remission. Vedolizumab showed slower onset (median 19 days to response) but led to clinical remission in 90.9% (Figure 4 and Table 5 and Table 6). The median duration of steroid treatment was numerically longer in the group requiring biologicals (median 114 vs 60.5 days), although this did not meet significance (*p* = 0.078) (Figure S3). We observed a higher need for biologicals in patients receiving combination ICI therapy versus anti-PD1/PDL-1 monotherapy (*p* = 0.011) (Figure 5). Patients with chronic/IBD-like pattern colitis mostly required treatment with biologicals (7/13) while those with lymphocytic-type colitis did not require any (0/6) (Table S4). A detailed overview of patients treated with biologicals can be found in Tables S5 and S6.

These findings indicate that both agents are effective, with infliximab associated with more rapid symptom control, while vedolizumab was linked to higher rates of clinical remission. The increased need for biologicals in patients receiving combination ICI treatment further supports its role as a predictor of steroid-refractory disease.

### 3.4. Rechallenge of ICIs

To investigate the safety of rechallenge of ICIs after colitis treatment, we assessed recurrence risk following rechallenge.

In 23/77 (29.9%) of patients, a rechallenge with an ICI was performed. The vast majority of patients (22/23) received PD-1/PD-L1 inhibitors, while one patient (1/23) received a combination of anti-CTLA-4 and anti-PD1 therapy. Relapse of colitis occurred in 4/23 rechallenged patients (17.4%) after a median time of 9.5 weeks (IQR 7.5–27.5 weeks) including the only patient treated with anti-PD1 + anti-CTLA-4 upon rechallenge. A detailed overview of these patients can be found in the Supplementary Materials (Table S7).

These findings suggest that ICI rechallenge is feasible in selected patients, with an acceptable recurrence risk.

### 3.5. Complications

To evaluate treatment-related risks, we assessed hospitalization and infection rates. We compared infection rates based on steroid duration, dosage, and the use of biologicals.

Median duration of follow-up since onset of ICI-induced colitis was 17.5 months (IQR 2.75–34 months).

Two thirds of patients (67.5%) required hospitalization (median stay 9 days; IQR 3.5–13 days). Severe infections necessitating admission occurred in 20.8% (Table S8) but were not associated with steroid duration (*p* = 0.382), dose (*p* = 0.967), or use of biologicals (*p* = 1). No deaths were directly attributed to colitis.

### 3.6. Oncological Outcomes

Finally, we examined whether immunosuppressive treatment influenced cancer outcomes.

**Figure 6:**
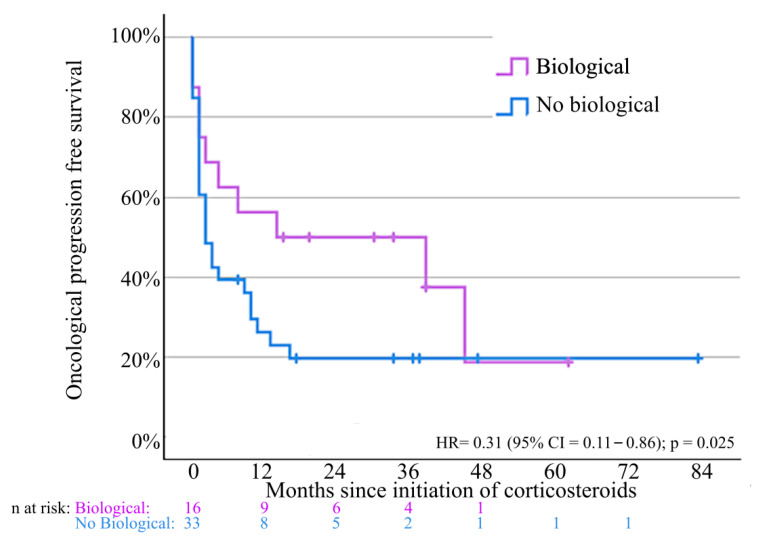
Progression-free survival for patients treated with systemic corticosteroids and biologicals compared to patients only treated with systemic corticosteroids. Cox regression analysis: hazard ratio for oncological disease progression = 0.31 (95% confidence interval = 0.11–0.86); *p* = 0.025. Not all patients could be included for survival analysis due to missing data.

Progressive oncological disease occurred in 73.6% of patients. Patients exposed to biologicals had significantly better PFS compared to corticosteroids alone (HR = 0.31; *p* = 0.025) (Figure 6).

**Figure 7:**
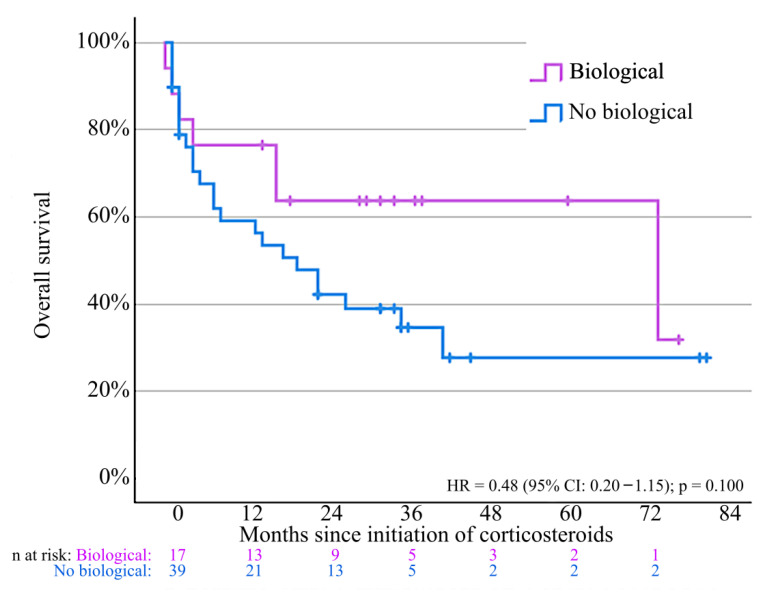
Overall survival for patients treated with systemic corticosteroids and biologicals compared to patients only treated with systemic corticosteroids. Cox regression analysis: hazard ratio for overall survival = 0.48 (95% confidence interval = 0.20–1.15); *p* = 0.100. Not all patients could be included for survival analysis due to missing data.

Overall mortality during follow-up was 56.0%, primarily due to progressive disease (71.4%). Overall survival was comparable for patients treated with biologicals and corticosteroids alone (Figure 7), although patients treated with vedolizumab showed higher survival compared to infliximab (*p* = 0.035) (Figure S4). Detailed causes of death are reported in Supplementary Table S9.

## 4. Discussion

In this retrospective observational study, we analyzed the clinical, endoscopic, histological, and therapeutic aspects of ICI-induced colitis in 77 patients. Combination immunotherapy with anti-PD-1/PD-L1 and anti-CTLA-4 agents was associated with more severe endoscopic inflammation and a higher need for biologicals, compared to anti-PD-1/PD-L1 monotherapy, but not with diarrhea severity. This supports that combination therapy predicts a corticosteroid-refractory course, underscoring the importance of early escalation to biologicals. Geukes Foppen et al. showed similar results with the grade of diarrhea not always reflecting the severity of endoscopic or histological inflammation, reaffirming the need for colonoscopy in all suspected cases, not only for diagnosis but also to anticipate treatment escalation [24].

Next, histological inflammation and/or increased apoptosis was present in the majority (88%) of patients with normal endoscopic findings (Mayo 0), highlighting the importance of random colonic biopsies in patients with suspected ICI-induced colitis. This finding directly addresses an important gap in current knowledge, as normal endoscopic appearance cannot reliably exclude colitis and may potentially misguide treatment decisions.

We observed that patients treated with biologicals had better PFS than those treated with corticosteroids alone. This is surprising and should be interpreted with caution, due to small sample size and possible confounders such as treatment burden or disease type, which were not assessed. A possible explanation is the more selective immunosuppression of biologicals, whereas corticosteroids are known to broadly suppress T-cell-mediated immunity and may thereby compromise antitumor effects of ICIs. However, treatment duration on corticosteroids did not differ between both groups. This observation is in contrast with a Dutch study in melanoma, where patients treated with second-line immunosuppressants had inferior PFS (HR 1.43, 95% CI, 1.07–1.90) and OS (HR 1.64, 95% CI, 1.16–2.32) [25]. Both infliximab and vedolizumab were effective as second-line immunosuppressants, with infliximab demonstrating a faster clinical and biochemical response, while vedolizumab had slower response rates. This finding is consistent with the retrospective study by Zou et al., and in line with ESMO guidelines recommending infliximab in severe cases requiring rapid symptom control [7,9,11,26]. Nevertheless, we did not observe reduced hospitalizations or shorter steroid exposure with vedolizumab, as previously reported [26]. The mortality rate was lower for patients treated with vedolizumab compared to infliximab; however, these results might be affected by the small sample size. Currently, two phase II trials are ongoing, one in Denmark and one in the United States, aiming to evaluate short-term efficacy and long-term safety of both agents [27,28].

Few studies have reported relapse rates of ICI-induced colitis after reintroduction of therapy. In our cohort, rechallenge was feasible in selected patients, with a low relapse rate (17.4%) and a good response to subsequent treatment. The relapse rate was lower than in other series, where rates up to 30–40% have been described [29]. This difference may reflect our careful patient selection for rechallenge, which should always be considered on a case-by-case basis after multidisciplinary consultation and shared decision-making [7,9]. The strengths of this study include the stringent enrolment of patients with colonic biopsies, providing a high level of diagnostic certainty and the integration of clinical, endoscopic, and histological data. All biopsies were reviewed by an expert pathologist on this topic. We provide real-world treatment outcomes, including both corticosteroid and biological regimens, and document the safety of rechallenge with ICIs in selected cases.

However, several limitations must be acknowledged. First, the retrospective design subjects the study to selection and documentation bias. Missing data were retrieved through cross-check of the electronic health record. There was no standardized follow-up or scheduled repeated endoscopy for all patients, which may have led to underestimation of endoscopic and biochemical remission rates. Second, our sample size was limited, reducing statistical power and the ability to detect significant associations or subgroup differences. We aimed to include all patients with histopathological confirmed colitis, treated in a tertiary care facility, where severe cases may be overrepresented.

In conclusion, this study confirms that ICI-induced colitis presents with heterogeneous clinical and pathological features and that endoscopic and histological evaluation are critical for diagnosis and treatment planning. Combination immunotherapy is associated with more severe colitis and a greater need for escalation to biologicals.

Given the increasing use of ICIs and the rising incidence of ICI-induced colitis, there is a need for prospective studies to compare the efficacy and safety of available second-line treatments. Until such data becomes available, individualized treatment based on severity, patient comorbidity, and multidisciplinary discussion remains essential. Our findings support the consideration of ICI rechallenge in selected patients, with close monitoring for recurrence of ICI-induced colitis.

## Figures and Tables

**Table 1:** Histopathological subtypes: definitions.

Histologic Patterns of Colitis	Definition
Active/infectious-type colitis pattern	This pattern often features focal inflammation, though it can also be prominent and diffuse. The focal form is often referred to as focal active colitis, while the diffuse form is known as diffuse acute colitis. In focal active colitis, only a few isolated foci of neutrophilic infiltration may be present.
Chronic/IBD-like colitis pattern	This pattern is usually characterized by mild signs of chronicity, such as basal plasmacytosis, without significant architectural distortion. However, in some cases—particularly those resistant to therapy—more pronounced chronic features resembling inflammatory bowel disease (IBD) may be observed. Chronic colitis can be either active or inactive.
Lymphocytic colitis pattern	Defined by an increased number of intraepithelial lymphocytes.
Collagenous colitis pattern	Characterized by a thickened subepithelial collagen layer.
Apoptosis	Increased apoptosis can be observed in the absence of active or significant lymphoplasmacytic inflammation in the lamina propria. As the histological findings do not align with any of the other three patterns, these cases are grouped under the active/infectious-type colitis pattern. Focal active inflammation is absent, although this may be due to sampling error. In some studies, this was classified as the apoptotic/GVHD colitis pattern. However, as our aim is to investigate the relationship between increased apoptosis and specific drug regimens, apoptosis is reported separately and the apoptotic colitis pattern category is discarded.

**Table 2:** Clinical, biochemical, and endoscopic response/remission: definitions.

Response/Remission	Definition
Clinical response	Decrease in symptom severity according to the treating physician.
Clinical remission	Complete resolution of symptoms according to the treating physician.
Biochemical response	A decrease in CRP of ≥50% or a decrease in calprotectin of ≥50% if fecal calprotectin was assessed.
Biochemical remission	Normalization of CRP (to values under the upper limit of normal) or fecal calprotectin < 250 µg/g if fecal calprotectin was assessed.
	If CRP or fecal calprotectin were below the lower limit of normal at time of diagnosis and treatment assessment, biochemical response was not assessable.
Endoscopic response	Mayo endoscopic subscore-1 level compared to the endoscopy at time of diagnosis. If the Mayo endoscopic subscore at time of diagnosis and evaluation was 0, endoscopic response was not assessable.
Endoscopic remission	Mayo endoscopic subscore = 0 upon repeated lower GI endoscopy.

**Table 3:** Baseline characteristics.

	ICI-Induced Colitis (*n* = 77)
Sex	
Men	44 (57.1%)
Women	33 (42.9%)
Median age, years	64 (31–87)
Type of tumor	
Melanoma	24 (31.2%)
Lung cancer	16 (20.8%)
Renal cell carcinoma	14 (18.2%)
Endometrial cancer	4 (5.2%)
Head and neck cancer	4 (5.2%)
Bladder cancer	3 (3.9%)
Cervic cancer	3 (3.9%)
Breast cancer	2 (2.6%)
Cholangiocarcinoma	2 (2.6%)
Hepatocellular carcinoma	1 (1.3%)
Cutaneous squamous cell carcinoma	1 (1.3%)
Ovarian cancer	1 (1.3%)
Gastric cancer	1 (1.3%)
Malignant pleural mesothelioma	1 (1.3%)
Type of immunotherapy	
Anti-PD-1/anti-PD-L1 monotherapy	28 (36.4%)
Anti-CTLA-4 and anti-PD-1/anti-PD-L1	18 (23.4%)
Anti-PD-1/anti-PD-L1 and chemotherapy	9 (11.7%)
Anti-PD-1/anti-PD-L1 and investigational drugs	8 (10.4%)
Anti-CTLA-4 monotherapy	7 (9.1%)
Anti-PD-1/anti-PD-L1 and anti-VEGF	2 (2.6%)
Anti-PD-1/anti-PD-L1 and TKI	2 (2.6%)
Anti-PD-1/anti-PD-L1 and anti-VEGF and chemotherapy	1 (1.3%)
Anti-CTLA-4 and anti-PD-1/anti-PD-L1 and chemotherapy	1 (1.3%)
Anti-CTLA-4 and anti-PD-1/anti-PD-L1 and investigational drugs	1 (1.3%)
Treatment setting	
Neo-adjuvant	2 (2.6%)
Adjuvant	8 (10.4%)
Locally advanced/metastatic	67 (87%)
Evaluation at diagnosis	
(1) Clinical: grade of diarrhea (*n* = 77)	
Grade 1	14 (18.2%)
Grade 2	38 (49.4%)
Grade 3	24 (31.2%)
Grade 4	1 (1.3%)
(2) Biochemical	
Calprotectin µg/g (*n* = 29), median (IQR)	323 (115–1055)
CRP mg/L (*n* = 74), median (IQR)	28.9 (7.68–78.6)
(3) Endoscopic findings (*n* = 74)	
Mayo 0	25 (33.8%)
Mayo 1	22 (29.7%)
Mayo 2	21 (28.4%)
Mayo 3	6 (8.1%)
(4) Histopathological findings (*n* = 74)	
Active/infectious-type colitis	48 (64.9%)
Chronic/IBD-type colitis	12 (16.2%)
Lymphocytic-type colitis	7 (9.5%)
Collagenous-type colitis	3 (4.1%)
Normal	4 (5.4%)
Presence of apoptosis	35 (47.3%)

**Table 4:** Distribution of treatment in first-, second-, and third-line for ICI-induced colitis.

1st Line Treatment	n (%)	2nd Line Treatment	n (%)	3rd Line Treatment	n (%)
Cs *	53 (68.8)	Cs	1 (1.3)	Infliximab	1 (1.3)
Cs + 5-ASA	1 (1.3)	Infliximab	7 (9.1)	Vedolizumab	4 (5.2%)
Cs + oral budesonide	1 (1.3)	Vedolizumab	8 (10.4)	No treatment needed	72 (93.5)
Cs + rectal budesonide	2 (2.6)	Vedolizumab + 5-ASA	1 (1.3)		
Cs + 5-ASA + oral budesonide	1 (1.3)	Vedolizumab + rectal budesonide	1 (1.3)		
5-ASA	1 (1.3)	5-ASA	1 (1.3)		
Oral budesonide	1 (1.3)	5-ASA + beclomethasone	1 (1.3)		
Beclomethasone	6 (7.8)	Oral budesonide	2 (2.6)		
No treatment needed	11 (14.3)	Rectal budesonide	1 (1.3)		
		Rectal budesonide + beclomethasone	1 (1.3)		
		No treatment needed	53 (68.8)		

* = Systemic corticosteroids.

**Table 5:** Clinical, biochemical, and endoscopic response/remission for patients treated with corticosteroids and/or biologicals.

Response/Remission	Clinical	Biochemical	Endoscopic
Systemic corticosteroids (overall) *	Response 79.6% (43/54) Remission 50% (27/54)	Response 51.9% (28/54) Remission 27.8% (15/54)	Response 41.7% (10/24) Remission 25% (6/24)
Infliximab	Response 100% (7/7) Remission 57.1% (4/7)	Response 85.7% (6/7) Remission 71.4% (5/7)	Response 66.7% (2/3) Remission 33.3% (1/3)
Vedolizumab	Response 100% (11/11) Remission 90.9% (10/11)	Response 50% (5/10) Remission 40% (4/10)	Response 90% (9/10) Remission 70% (7/10)
Vedolizumab and infliximab	*n* = 1, response (follow-up in another hospital)	Response	No response

* Biochemical and endoscopic evaluation was not assessable for 8/54 (14.8%) and 2/23 (8.7%) of patients (see definitions in Table 3). Clinical, biochemical, and endoscopic evaluation of response was not performed by the treating physician in, respectively, 4/59 (6.8%), 5/59 (8.5%), and 35/58 (59.3%) of our patients. One patient (1/59) had clinical remission before initiation of corticosteroids. These patients were not included for analysis.

**Table 6:** Time to clinical, biochemical, and endoscopic response/remission for patients treated with corticosteroids and/or biologicals.

Response/ Remission	Time to Clinical Response/ Remission	Time to biochemical Response/ Remission	Time to endoscopic Response/ Remission
Systemic corticosteroids	Response: *n* = 43, median: 13 days Remission: *n* = 27, median: 17.5 days	Response: *n* = 28, median: 13 days Remission: *n* = 15, median: 14 days	Response: *n* = 10, median: 54 days Remission: *n* = 6, median: 65 days
			
Infliximab	Response: *n* = 7, median: 7 days	Response: *n* = 6, median: 9.5 days	Response: *n* = 2, median: 71 days
	Remission: *n* = 4, median: 12 days	Remission: *n* = 5, median: 14 days	Remission: *n* = 1, mean: 61 days
			
Vedolizumab	Response: *n* = 11, median: 19 days	Response: *n* = 5, median: 53 days	Response: *n* = 9, median: 52 days
	Remission: *n* = 10, median: 50.5 days	Remission: *n* = 4, median: 52.5 days	Remission: *n* = 7, median: 53 days
			
Vedolizumab and infliximab	Response: *n* = 1, median: 20 days	Response: *n* = 1, median: 8 days	No response

## Data Availability

Dataset available on request from the authors. The dataset underlying this study is not publicly available due to its involvement in ongoing research.

## Supplementary Materials

The following supporting information can be downloaded at https://www.mdpi.com/article/10.3390/jcm14207219/s1; List of all extracted variables for analysis; Further description of investigational immunomodulatory drugs; Overview of all hypothesis tests performed; Supplementary findings; Figure S1: Box plot for distribution of fecal calprotectin according to histologic pattern of ICI-induced colitis; Figure S2: Box plot for distribution of CRP according to histological pattern of ICI-induced colitis; Figure S3: Duration of systemic corticosteroids by colitis treatment regimen (corticosteroids vs biologicals); Figure S4: Overall survival for patients treated with vedolizumab compared to patients treated with infliximab; Table S1: Grade of diarrhea along the Common Terminology Criteria for Adverse Events (CTCAE) v5.01 scale; Table S2: Mayo endoscopic subscore; Table S3: Other irAEs at diagnosis of ICI-induced colitis; Table S4: Usage of biologicals per histopathological subtype; Table S5: Overview of patients treated with infliximab; Table S6: Overview of patients treated with vedolizumab; Table S7: Overview of patients with a relapse of ICI-induced colitis after rechallenge; Table S8: Infections leading to hospitalization; Table S9: Causes of death.

## Abbreviations

The following abbreviations are used in this manuscript:

ICI	Immune checkpoint inhibitor
CTLA-4	Cytotoxic T-lymphocyte antigen 4
LAG-3	Lymphocyte activating gene 3
PD-1	Programmed cell death 1
PD-L1	Programmed cell death ligand 1
GI	Gastro-intestinal
irAE	Immune-related adverse event
CTCAE	Common Terminology Criteria for Adverse Events
TNFα	Tumor necrosis factor alpha
IBD	Inflammatory bowel disease
CRP	C-reactive protein
5-ASA	5-aminosalicylic acid
