# Supplementary material for: Immune Checkpoint-Induced Colitis: A Single-Center Retrospective Cohort Study
Source: J Clin Med. 2025 Oct 13;14(20):7219. doi: 10.3390/jcm14207219 (PMC12565555; doi:10.3390/jcm14207219)
Supplement: Supplementary file 1 [file jcm-14-07219-s001.zip › jcm-3852213-supplementary.pdf]

Supplementary Material

Table of contents

List of all extracted variables for analysis 2

Further description of investigational immunomodulatory drugs 3

Overview of all hypothesis tests performed 3

Supplementary findings 5

Supplementary figures 6

Figure S1: Box plot for distribution of faecal calprotectin according to histologic pattern of ICI-induced colitis. 6

Figure S2: Box plot for distribution of CRP according to histological pattern of ICI-induced colitis. 6

Figure S3: Duration of systemic corticosteroids by colitis treatment regimen (corticosteroids vs biologicals) 7

Figure S4: Overall survival for patients treated with vedolizumab compared to patients treated with infliximab. 8

Supplementary tables 9

Table S1: Grade of diarrhoea along the Common Terminology Criteria for Adverse Events (CTCAE) v5.01 scale. 9

Table S2: Mayo endoscopic subscore 9

Table S3: Other irAEs at diagnosis of ICI-induced colitis 9

Table S4: Usage of biologicals per histopathological subtype. 9

Table S5: Overview of patients treated with infliximab. 1

Table S6: Overview of patients treated with vedolizumab. 3

Table S7: Overview of patients with a relapse of ICI-induced colitis after rechallenge. 6

Table S8: Infections leading to hospitalization. 6

Table S9: Causes of death. 9

List of all extracted variables for analysis

Demographic data

Gender, birth year, weight, height, smoking status.

Comorbidities

Charleston comorbidity index (age, history of myocardial infarction, history of chronic heart failure, history of peripheral vascular disease, history of CVA or TIA, history of dementia, history of COPD, history of connective tissue disease, history of peptic ulcer disease, history of liver disease, history of diabetes mellitus, history of hemiplegia, history of moderate to severe chronic kidney disease, history of solid tumour other than the tumours for which the ICIs are given, history of leukaemia, history of lymphoma, history of AIDS), history of auto-immune disease, history of inflammatory bowel disease, subject to immune suppressive drugs.

Characteristics of the tumour

Tumour type, date of tumour diagnosis, TNM classification at time of starting ICIs, tumour stage at time of starting ICIs.

Characteristics of immunotherapy regimen

Type of immune checkpoint inhibitor, co-administration of chemotherapy, co-administration of anti-VEGF monoclonal antibodies, co-administration of tyrosine kinase inhibitors, co-administration of investigational immunomodulatory drugs, line of treatment for malignancy, use of systemic anticancer therapy in the last three months (if yes, which?), history of exposition to ICIs in the past (if yes, which?), history of immune-related adverse events (if yes, which?), treatment setting (neo-adjuvant, adjuvant, metastatic/locally advanced), date of start of ICIs, discontinuation of ICIs (if yes, date of discontinuation of ICIs and reason for discontinuation of ICIs).

Evaluation of oncological outcomes

First oncological outcome after discontinuation of ICIs (complete remission, partial remission, stable disease, progressive disease as defined by RECIST 1.1), date of CT scan for first oncological evaluation after discontinuation of ICIs, presence of progressive disease as defined by RECIST 1.1 (if yes, date of progressive disease).

Evaluation of ICI-induced colitis

Presence of ICI-induced colitis, date of onset of colitis, grade of colitis (CTCAE criteria v.5.0), grade of diarrhoea (CTCAE criteria v.5.0), presence of other immune-related adverse events (if yes, which?), date of lower endoscopy, type of lower endoscopy (sigmoidoscopy or ileocolonoscopy), treatment with corticosteroids at time of endoscopy, Mayo endoscopic subscore, histological pattern of colitis (Active/infectious-type colitis pattern, chronic colitis pattern, lymphocytic colitis pattern, collagenous colitis pattern), increase in apoptosis, test result for cytomegalovirus immunohistochemistry on colon biopsy, white blood cell count (/µl), neutrophil count (/µl), lymphocyte count (/µl), value of c-reactive protein (mg/l), value of faecal calprotectin (mg/kg), date of c-reactive protein, date of faecal calprotectin, presence of positive faecal culture. These variables were repeated for each treatment line for ICI-induced colitis.

Treatment of ICI-induced colitis

Line of treatment (number), treatment with 5-ASA, treatment with oral budesonide, treatment with rectal budesonide, treatment with oral beclomethasone, treatment with systemic corticosteroids (if yes, type of drug (methylprednisolone, prednisolone, hydrocortisone, dexamethasone), dose of drug (mg), mode of administration (oral or intravenous), start date and stop date), restart with systemic corticosteroids (if yes, type of drug (methylprednisolone, prednisolone, hydrocortisone, dexamethasone), dose of drug (mg), mode of administration (oral or intravenous), start date, stop date), treatment with infliximab (if yes, dose of drug (mg), total amount of infusions, start date, stop date), treatment with vedolizumab (if yes, dose of drug (mg), total amount of infusions, start date, stop date), other drugs used (if yes, specify). These variables were repeated for each treatment line for ICI-induced colitis.

Evaluation of treatment response

Physician's assessment (remission, response, no response, not assessable), date of assessment, value of c-reactive protein (mg/l), date of c-reactive protein assessment, value of faecal calprotectin (mg/kg), date of faecal calprotectin assessment, performance of endoscopic evaluation (if yes, date of endoscopy, Mayo endoscopic subscore, physician's assessment at time of endoscopy). These variables were repeated for each treatment line for ICI-induced colitis.

Rechallenge of ICIs

Type of immune checkpoint inhibitor, co-administration of chemotherapy, co-administration of anti-VEGF monoclonal antibodies, co-administration of tyrosine kinase inhibitors, co-administration of investigational immunomodulatory drugs, start date of rechallenge of ICIs, discontinuation of rechallenge of ICIs (if yes, date of last administration, reason for discontinuation). These variables were repeated for each treatment line for ICI-induced colitis.

Relapse of colitis

Date of relapse, grade of colitis (CTCAE criteria v.5.0), grade of diarrhoea (CTCAE criteria v.5.0), treatment with ICIs at time of colitis, value of c-reactive protein (mg/l), value of faecal calprotectin (mg/kg), date of c-reactive protein assessment, date of faecal calprotectin assessment, performance of endoscopic evaluation (if yes, date of endoscopy, Mayo endoscopic subscore), histological pattern of colitis (active/infectious-type colitis pattern, chronic colitis pattern, lymphocytic colitis pattern, collagenous colitis pattern), increase in apoptosis, test result for cytomegalovirus immunohistochemistry on colon biopsy, treatment of ICI-induced colitis (oral budesonide, rectal budesonide, oral beclomethasone, oral corticosteroids, intravenous corticosteroids, infliximab, vedolizumab), evaluation of treatment response: physician's assessment (remission, response, no response, not assessable), date of assessment, value of c-reactive protein (mg/l), value of faecal calprotectin (mg/kg), date of c-reactive protein assessment, date of faecal calprotectin assessment.

Adverse events and mortality

Hospitalization required due to colitis (if yes, date of admission, date of discharge), second hospitalization required due to colitis (if yes, date of admission, date of discharge), third hospitalization required due to colitis (if yes, date of admission, date of discharge), infection leading to hospitalization (if yes, specify), other immune-related adverse event leading to hospitalization (if yes, specify), mortality during follow-up (if yes, reason of death, date of decease), date of last follow-up.

Further description of investigational immunomodulatory drugs

Nine patients received an ICI in combination with an investigational immunomodulatory drug. This was performed as part of a clinical trial. Of these patients, two received an anti-IL8 drug [26], one received L19IL2 (Darleukin ©) and one venetoclax [27, 28]. The other five received radiotherapy of a tumour site and a cocktail consisting of low-dose cyclophosphamide, aspirin, lansoprazole, vitamin D, and curcumin [29].

Overview of all hypothesis tests performed

Relation between variables "type immunotherapy" (anti-PD-1/PDL-1 with anti-CTLA-4 versus anti-PD-1/PDL-1 monotherapy) and "Mayo endoscopic subscore": Chi^2 test ($p = 0.029$).
Relation between variables "type immunotherapy" (anti-PD-1/PDL-1 with anti-CTLA-4 versus anti-PD-1/PDL-1 monotherapy versus anti-CTLA-4 monotherapy) and "Mayo endoscopic subscore": Chi^2 test ($p = 0.121$).
Relation between variables "histopathological pattern of colitis" (active/infectious-type colitis pattern versus chronic colitis pattern versus collagenous colitis pattern versus lymphocytic colitis pattern) and "CRP": Kruskal–Wallis test ($p = 0.295$).
Relation between variables "histopathological pattern of colitis" (active/infectious-type colitis pattern versus chronic colitis pattern and collagenous colitis pattern and lymphocytic colitis pattern) and "CRP": Mann–Whitney U test ($p = 0.100$).
Relation between variables "histopathological pattern of colitis" (active/infectious-type colitis pattern versus chronic colitis pattern versus collagenous colitis pattern versus lymphocytic colitis pattern) and "faecal calprotectin": Mann–Whitney U test ($p = 0{,}011$).

Relation between variables “histopathological pattern of colitis” (chronic colitis pattern versus active/infectious-type and collagenous colitis pattern and lymphocytic colitis pattern) and “faecal calprotectin”: Mann–Whitney U test (p = 0,002).
Relation between variables “grade of diarrhoea” and “Mayo endoscopic subscore”: Chi² test (p = 0,101).
Relation between variables “grade of diarrhoea” and “type immunotherapy” (anti-PD-1/PDL-1 with anti-CTLA-4 versus anti-PD-1/PDL-1 monotherapy versus anti-CTLA-4 monotherapy): Chi² test (p = 0.261).
Relation between variables “grade of diarrhoea” and “type immunotherapy” (anti-PD-1/PDL-1 with anti-CTLA-4 versus anti-PD-1/PDL-1 monotherapy): Fisher’s Exact test (p = 0.169), Chi² test (p = 0.165).
Relation between variables “Mayo endoscopic subscore” and “histopathological type” (active/infectious type versus chronic/IBD type versus collagenous type versus lymphocytic type): Chi² test (p = 0.165).
Relation between variables “Grade of diarrhoea” and “histopathological type” (active/infectious type versus chronic/IBD type versus collagenous type versus lymphocytic type): Chi² test (p = 0.199).
Relation between variables “type immunotherapy” (anti-PD-1/PDL-1 with anti-CTLA-4 versus anti-PD-1/PDL-1 monotherapy versus anti-CTLA-4 monotherapy) and “histopathological pattern of colitis” (active/infectious-type colitis pattern versus chronic/IBD colitis pattern versus collagenous colitis pattern versus lymphocytic colitis pattern): Chi² test (p = 0.415).
Relation between variables “type immunotherapy” (anti-PD-1/PDL-1 monotherapy versus anti-CTLA-4 monotherapy) and “histopathological pattern of colitis” (active/infectious-type colitis pattern versus chronic/IBD colitis pattern versus collagenous colitis pattern versus lymphocytic colitis pattern) : Chi² test (p = 0.746).
Relation between variables “type immunotherapy” (anti-PD-1/PDL-1 with anti-CTLA-4 versus anti-PD-1/PDL-1 monotherapy) and “histopathological pattern of colitis” (active/infectious-type colitis pattern versus chronic/IBD colitis pattern versus collagenous colitis pattern versus lymphocytic colitis pattern) : Chi² test (p = 0.159).
Relation between variables “increase in apoptosis” and “Mayo endoscopic subscore”: Chi² test (p = 0.937).
Relation between variables “increase in apoptosis” and “grade of diarrhoea”: Chi² test (p = 0.137).
Relation between variables “increase in apoptosis” and “CRP”: Mann–Whitney U test (p = 0.272).
Relation between variables “increase apoptosis” and “faecal calprotectin”: Mann–Whitney U test (p = 0.161).
Relation between variables “increase in apoptosis” and “type immunotherapy” (anti-PD-1/PDL-1 with anti-CTLA-4 versus anti-PD-1/PDL-1 monotherapy versus anti-CTLA-4 monotherapy): Chi² test (p = 0.539).
Relation between variables “increase in apoptosis” and “type immunotherapy” (anti-PD-1/PDL-1 monotherapy versus anti-CTLA-4 monotherapy): Fisher’s Exact test (p = 0.669), Chi² test (p = 0.609).
Relation between variables “increase in apoptosis” and “type immunotherapy” (anti-PD-1/PDL-1 with anti-CTLA-4 versus anti-PD-1/PDL-1 monotherapy): Fisher’s Exact test (p = 0.789), Chi² test (p = 0.661).
Correlation between variables “systemic corticosteroid starting dose” and “duration of treatment with corticosteroids”: Spearman correlation coefficient (p = 0.857).
Correlation between variables “duration between start of symptoms of ICI-induced colitis and initiation of systemic corticosteroids” and “duration of treatment with corticosteroids”: Spearman correlation coefficient (p = 0.362).
Relation between variables “grade of diarrhoea” and “duration of treatment with corticosteroids”: Kruskal–Wallis test (p = 0.965).
Relation between variables “Mayo endoscopic subscore” and “duration of treatment with corticosteroids”: Kruskal–Wallis test (p = 0.109).
Relation between variables “Type of colitis treatment (biologicals vs. only systemic corticosteroids” and “duration of treatment with corticosteroids”: Mann–Whitney U test (p = 0.078).
Relation between variables “type immunotherapy” (anti-PD-1/PDL-1 with anti-CTLA-4 versus anti-PD-1/PDL-1 monotherapy versus anti-CTLA-4 monotherapy) and “duration of treatment with corticosteroids”: Kruskal–Wallis test (p = 0.137).
Relation between variables “type immunotherapy” (anti-PD-1/PDL-1 with anti-CTLA-4 versus anti-PD-1/PDL-1 monotherapy) and “duration of treatment with corticosteroids”: Kruskal–Wallis test (p = 0.143).
Relation between variables “type immunotherapy (without investigational drugs)” (anti-PD-1/PDL-1 with anti-CTLA-4 versus anti-PD-1/PDL-1 monotherapy versus anti-CTLA-4 monotherapy) and “duration of treatment with corticosteroids”: Kruskal–Wallis test (p = 0.349).

Relation between variables "type immunotherapy (without investigational drugs)" (anti-PD-1/PDL-1 with anti-CTLA-4 versus anti-PD-1/PDL-1 monotherapy) and "duration of treatment with corticosteroids": Kruskal–Wallis test (p = 0.316).

Relation between variables "use of biologicals" and "type immunotherapy" (anti-PD-1/PDL-1 with anti-CTLA-4 versus anti-PD-1/PDL-1 monotherapy versus anti-CTLA-4 monotherapy): Chi² (p = 0.015).

Relation between variables "use of biologicals" and "type immunotherapy" (anti-PD-1/PDL-1 with anti-CTLA-4 versus anti-PD-1/PDL-1 monotherapy or anti-CTLA-4 monotherapy): Fisher's Exact test (p = 0.032), Chi² (p = 0.016).

Relation between variables "use of biologicals" and "type immunotherapy" (anti-PD-1/PDL-1 with anti-CTLA-4 versus anti-PD-1/PDL-1 monotherapy): Fisher's Exact test (p = 0.011), Chi² (p = 0.006).

Relation between variables "use of biologicals" and "histopathological pattern of colitis" (chronic/IBD colitis pattern versus active/infectious-type colitis pattern and collagenous colitis pattern and lymphocytic colitis pattern) : Fisher's Exact test (p = 0.014).

Relation between variables "grade of diarrhoea" and "type immunotherapy" (anti-PD-1/PDL-1 with anti-CTLA-4 versus anti-PD-1/PDL-1 monotherapy versus anti-CTLA-4 monotherapy): Chi² (p = 0.862).

Relation between variables "Mayo endoscopic subscore" and "type immunotherapy" (anti-PD-1/PDL-1 with anti-CTLA-4 versus anti-PD-1/PDL-1 monotherapy versus anti-CTLA-4 monotherapy): Chi² (p = 0.093).

Relation between variables "Type of biological" (infliximab versus vedolizumab) and "duration of treatment with systemic corticosteroids": Mann–Whitney U test (p = 0.897).

Correlation between "duration of treatment with systemic corticosteroids" and "duration of hospitalization: Spearman correlation coefficient (p = 0.669).

Relation between "type of biological" (infliximab versus vedolizumab) and "duration of hospitalization": Mann–Whitney U test (p = 0.243).

Relation between "use of biologicals" and "duration of hospitalization": Mann–Whitney U test (p = 0.282).

Relation between "duration of treatment with systemic corticosteroids" and "need for hospitalization due to infection": Mann–Whitney U test (p = 0.382).

Relation between "highest dose of systemic corticosteroids" and "need for hospitalization due to infection": Mann–Whitney U test (p = 0.882).

Relation between "use of biologicals" and "need for hospitalization due to infection" in patients treated with corticosteroids: Fisher's Exact test (p = 0.987).

Survival analysis for "oncological progressive disease" and "use of biologicals" in patients treated with corticosteroids: Cox regression analysis (p = 0.025)

Survival analysis for "mortality" according to "use of biological" in patients treated with systemic corticosteroids: Cox regression analysis (p = 0.100).

Survival analysis for "oncological progressive disease" according to "type of biological" (infliximab versus vedolizumab) in patients treated with systemic corticosteroids: Cox regression analysis (p = 0.080).

Survival analysis for "mortality" according to "type of biological" (infliximab versus vedolizumab) in patients treated with systemic corticosteroids: Cox regression analysis (p = 0.035).

Supplementary findings

Levels of faecal calprotectin were higher in patients with chronic/IBD-type colitis (p = 0,002):

Faecal calprotectin in chronic/IBD-type: median = 2031 μg/g (IQR = 1055 – 3531,5).

Faecal calprotectin in active/infectious-type: median = 304 μg/g (IQR = 127 – 914).

Faecal calprotectin in collagenous-type: median = 323 μg/g (n too small for IQR).

Faecal calprotectin in lymphocytic-type: median = 111 μg/g (n too small for IQR).

No significant differences were found for CRP between the different histological patterns (p = 0.295):

CRP in active/infectious type: median = 36,6 mg/L (IQR = 8,2 – 96,8).

CRP in chronic/IBD type: median = 46,7 mg/L (IQR = 3,3 – 74,8).

CRP in lymphocytic type: median = 14,4 mg/L (IQR = 2,2 – 18,8).

CRP in collagenous type: median = 10,4 mg/L (n too small for IQR).

No significant correlation was observed between the grade of diarrhoea and the Mayo endoscopic subscore (p = 0.101). There was no significant difference for Mayo endoscopic subscore (p = 0.090), grade of diarrhoea (p = 0.566) and type of ICI regimen (p = 0.220) between the different histological patterns of colitis.

Supplementary figures

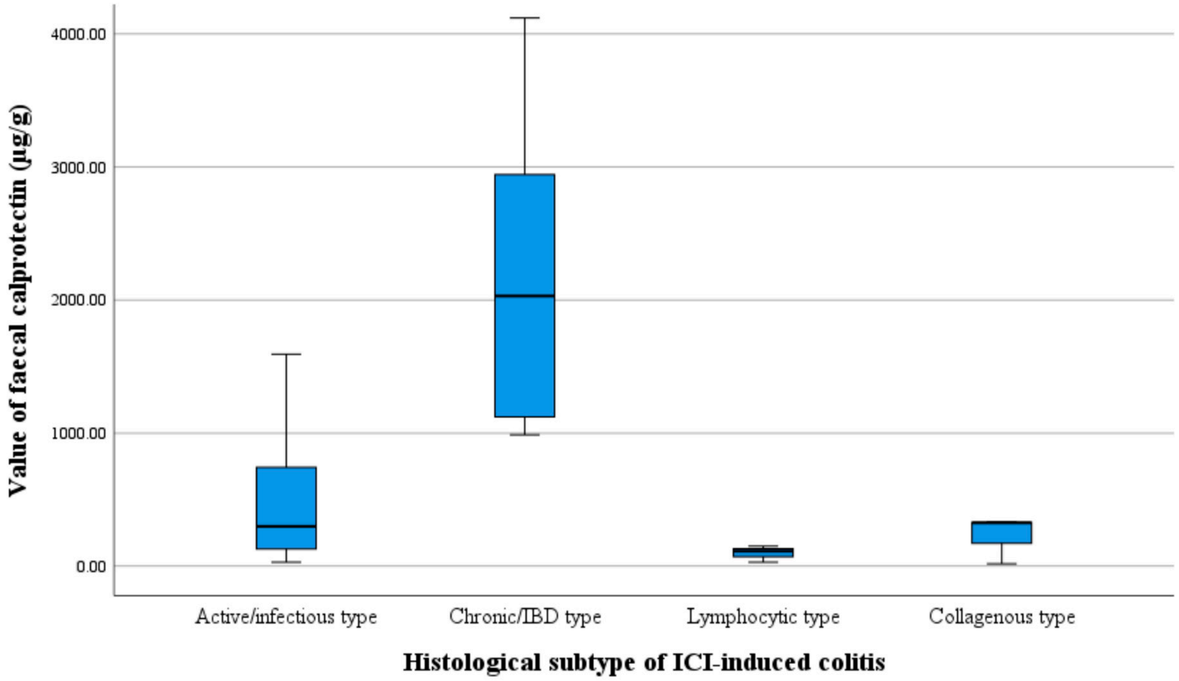

Figure S1. Box plot for distribution of faecal calprotectin according to histologic pattern of ICI-induced colitis.

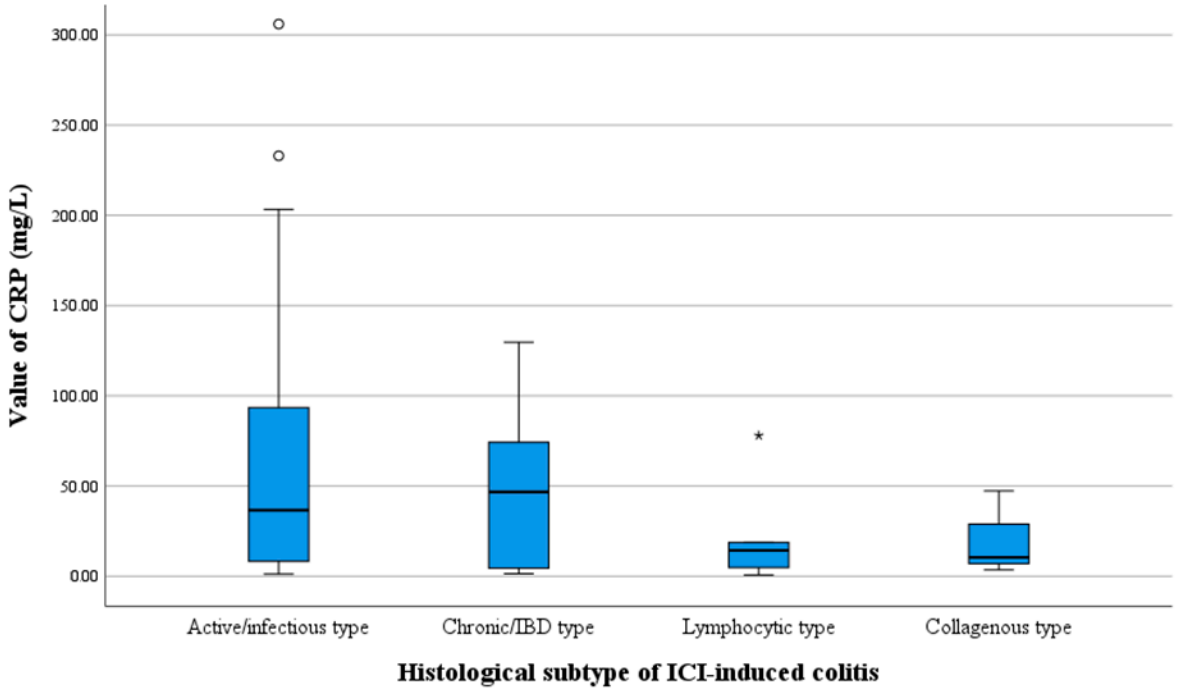

Figure S2. Box plot for distribution of CRP according to histological pattern of ICI-induced colitis.

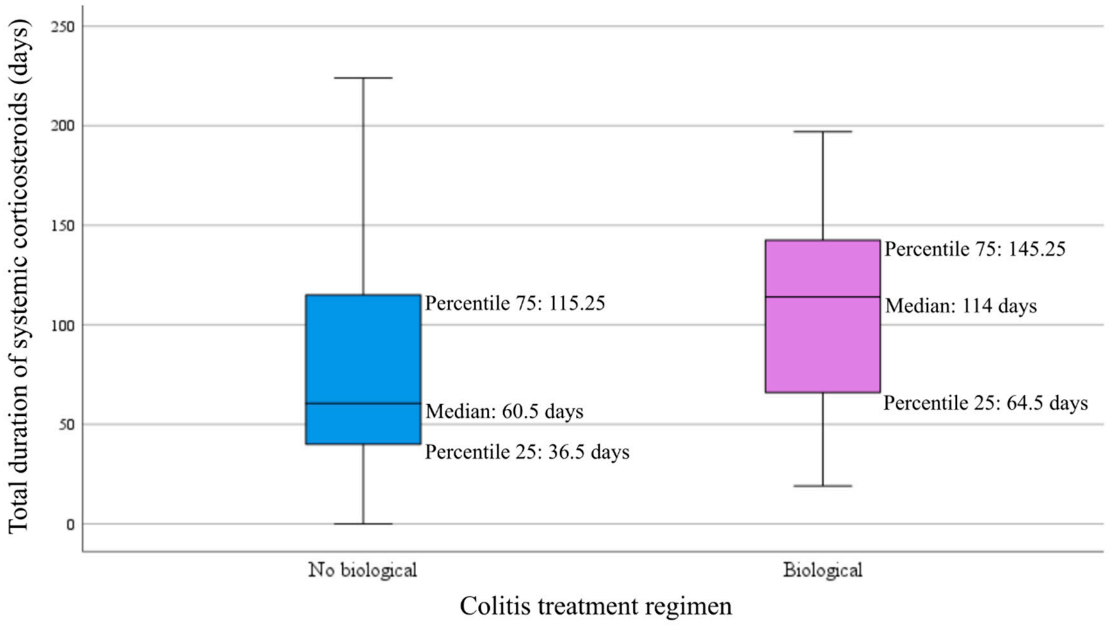

Figure S3. Duration of systemic corticosteroids by colitis treatment regimen (corticosteroids vs. biologicals).

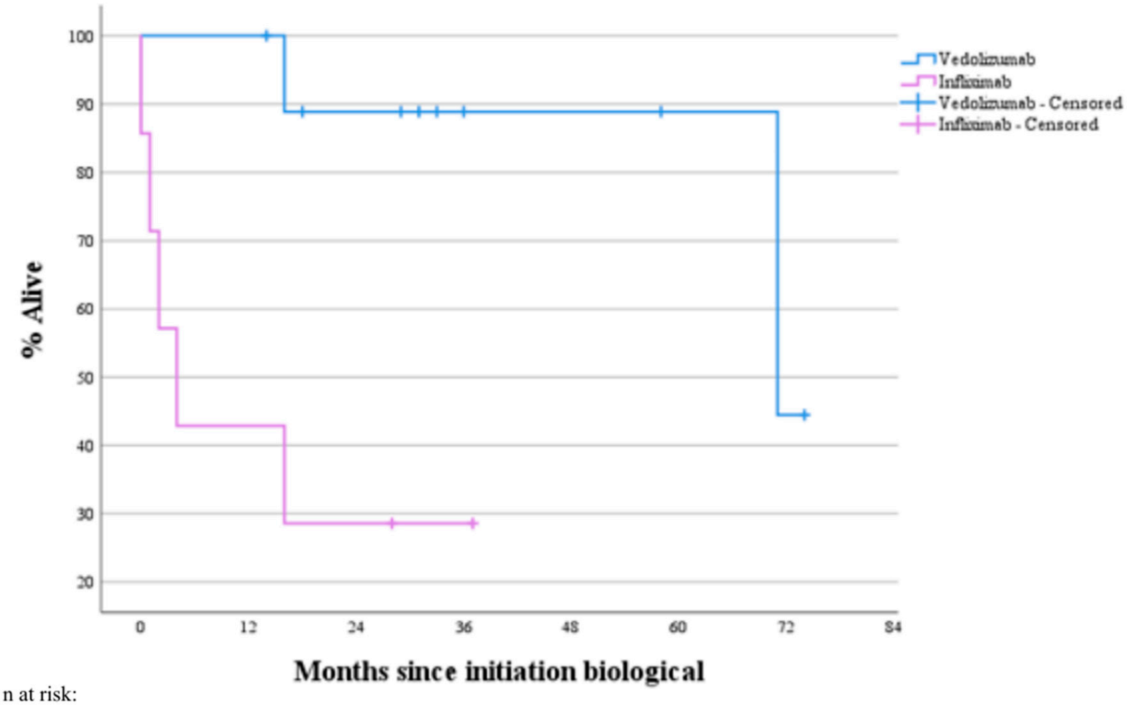

n at risk:

Vedolizumab:	10	10	7	4	3	2	1
Infliximab:	6	2	1				

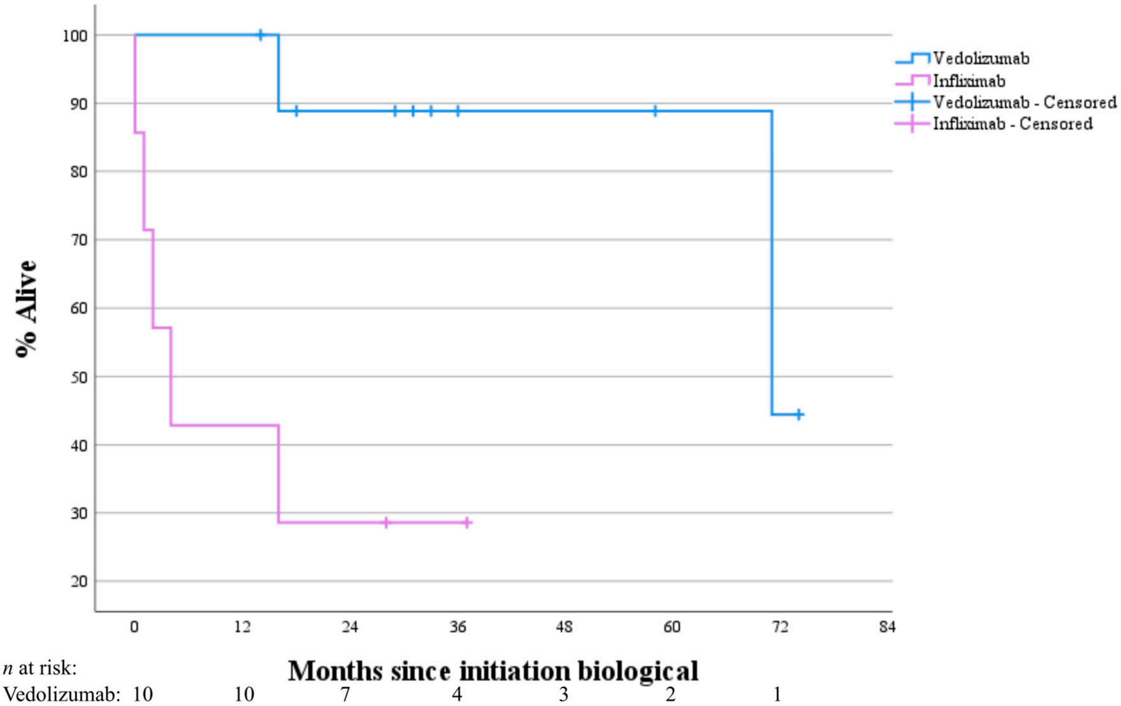

n at risk:

Vedolizumab:	10	10	7	4	3	2	1
Infliximab:	6	2	1				

Figure S4: Overall survival for patients treated with vedolizumab compared to patients treated with infliximab. Cox regression analysis: hazard ratio for death = 0.095 (95% confidence interval = 0.01 – 0.84); p = 0.035.

Supplementary tables

Table S1. Grade of diarrhoea along the Common Terminology Criteria for Adverse Events (CTCAE) v5.01 scale.

	Grade 1	Grade 2	Grade 3	Grade 4	Grade 5
**Diarrhoea**	Increase of <4 stools per day over baseline, mild increase in ostomy output compared to baseline	Increase of 4 - 6 stools per day over baseline, moderate increase in ostomy output compared to baseline, limiting instrumental ADL	Increase of >=7 stools per day over baseline, hospitalization indicated, severe increase in ostomy output compared to baseline, limiting self care ADL	Life-threatening consequences, urgent intervention indicated	Death

Table S2. Mayo endoscopic subscore.

Score	Disease activity	Endoscopic features
**0**	Normal or inactive	None
**1**	Mild	Erythema, decreased vascular pattern, mild friability
**2**	Moderate	Marked erythema, absent vascular pattern, friability, erosions
**3**	Severe	Spontaneous bleeding, ulcerations

Table S3. Other irAEs at diagnosis of ICI-induced colitis.

Other irAE	Upper GI	Pneumonitis	Skin toxicity	Nephritis	Hepatitis	Endocrine
	10	2	3	3	5	6

Table S4. Usage of biologicals per histopathological subtype.

		Use of biological		Total
		No	Yes	
Type colitis	Acute/infectious type	41	9	50
	Chronic type	6	7	13
	Lymphocytic type	6	0	6*
	Collagenous type	1	2	3
	Normal	3	1	4
Total		57	19	76*

*One patient with lymphocytic-type colitis was excluded from analysis because the patient received infliximab for irAE gastritis.

Table S5. Overview of patients treated with infliximab.

	Type ICI	Evaluation at diagnosis	First line treatment	Evaluation response	Second line treatment	Evaluation response	Third line treatment	Evaluation response
**Case 1**	Anti-CTLA-4 and anti-PD-1/PD-L1	Clinical: grade 3 diarrhea<br>Biochemical: CRP 147.6 mg/L, calprotectin 1593 µg/g<br>Endoscopic: Mayo 2<br>Anatomopathology: active/infectious type colitis, presence of apoptosis	Systemic corticosteroids	Clinical: no response<br>Biochemical: response<br>Endoscopic: not evaluated	Infliximab (2 infusions)	Clinical: response, no follow-up because of †<br>Biochemical: remission<br>Endoscopic: not evaluated	/	/
**Case 2**	Anti-CTLA-4 and anti-PD-1/PD-L1	Clinical: grade 2 diarrhea<br>Biochemical: CRP 46.7 mg/L, calprotectin 2031 µg/g<br>Endoscopic: Mayo 2<br>Anatomopathology: chronic/IBD-like type colitis	Systemic corticosteroids	Clinical: response (but still diarrhea 5-6/day)<br>Biochemical: remission<br>Endoscopic: not evaluated	Vedolizumab (1 infusion)	Clinical: no response<br>Biochemical: no response<br>Endoscopic: no response	Infliximab (3 infusions)	Clinical: response<br>Biochemical: response<br>Endoscopic: no response (further follow-up in another hospital)
**Case 3**	Anti-CTLA-4	Clinical: grade 2 diarrhoea<br>Biochemical: CRP 49.3 mg/L, calprotectin 4120 µg/g<br>Endoscopic: Mayo 2<br>Anatomopathology: chronic/IBD-like type colitis, presence of apoptosis	Systemic corticosteroids	Clinical: no response<br>Biochemical: response<br>Endoscopic: no response	Infliximab (3 infusions)	Clinical: remission<br>Biochemical: remission<br>Endoscopic: remission	/	/

**Case 4**	Anti-PD-1/PD-L1	Clinical: grade 3 diarrhea<br>Biochemical: CRP 31 mg/L, calprotectin /<br>Endoscopic: Mayo 1<br>Anatomopathology: chronic/IBD-like type colitis	Rectal budesonide and systemic corticosteroids	Clinical: no responseBiochemical: response<br>Endoscopic: no response	Infliximab (2 infusions)	Clinical: remission<br>Biochemical: remission<br>Endoscopic: not evaluated	/	/
**Case 5**	Anti-PD-1/PD-L1 and anti-VEGF	Clinical: grade 3 diarrhea<br>Biochemical: CRP 42.2 mg/L, calprotectin /<br>Endoscopic: Mayo 1<br>Anatomopathology: active/infectious type colitis, presence of apoptosis	Systemic corticosteroids	Clinical: remissionBiochemical: no response<br>Endoscopic: no response	Infliximab (1 infusion)	Clinical: response, no follow-up because of †Biochemical: no response<br>Endoscopic: no response	/	/
**Case 6**	Anti-CTLA-4 and anti-PD-1/PD-L1	Clinical: grade 3 diarrhea<br>Biochemical: CRP 140 mg/L, calprotectin /<br>Endoscopic: Mayo 2<br>Anatomopathology: active/infectious type colitis	Systemic corticosteroids	Clinical: no responseBiochemical: no response<br>Endoscopic: not evaluated	Infliximab (1 infusion)	Clinical: response, no follow-up because of †Biochemical: response<br>Endoscopic: not evaluated	/	/
**Case 7**	Anti-CTLA-4 and anti-PD-1/PD-L1	Clinical: grade 2 diarrhea<br>Biochemical: CRP 73.7 mg/L, calprotectin 2943 µg/g<br>Endoscopic: Mayo 3<br>Anatomopathology: chronic/IBD-like type colitis, presence of apoptosis	Systemic corticosteroids	Clinical: no responseBiochemical: response<br>Endoscopic: no response	Infliximab (3 infusions)	Clinical: remission<br>Biochemical: remission<br>Endoscopic: not evaluated	/	/

**Case 8**	Anti-CTLA-4	Clinical: grade 2 diarrhea<br>Biochemical: CRP 123.4 mg/L, calprotectin 940 µg/g<br>Endoscopic: not evaluated<br>Anatomopathology: active/infectious type colitis	Systemic corticosteroids	Clinical: no responseBiochemical: remission<br>Endoscopic: not assessable	Infliximab (3 infusions)	Clinical: remissionBiochemical: remission<br>Endoscopic: response	/	/

Table S6. Overview of patients treated with vedolizumab.

	**Type ICI**	**Evaluation at diagnosis**	**First line treatment**	**Evaluation response**	**Second line treatment**	**Evaluation response**	**Third line treatment**	**Evaluation response**
**Case 1**	Anti-PD-1/anti-PD-L1 and anti-VEGF and chemotherapy	Clinical: grade 2 diarrhea<br>Biochemical: CRP 2.5 mg/L, calprotectin 151 µg/g<br>Endoscopic: Mayo 1<br>Anatomopathology: active/infectious type colitis, presence of apoptosis	Systemic corticosteroids	Clinical: response<br>Biochemical: not assessable<br>Endoscopic: no response	Vedolizumab (5 infusions)	Clinical: remission<br>Biochemical: remission<br>Endoscopic: remission	/	/
**Case 2**	Anti-CTLA-4 and anti-PD-1/PD-L1	Clinical: grade 1 diarrhea<br>Biochemical: CRP 5.6 mg/L, calprotectin 988 µg/g<br>Endoscopic: Mayo 2<br>Anatomopathology: chronic/IBD-like type colitis	Systemic corticosteroids	Clinical: response<br>Biochemical: remission<br>Endoscopic: no response	Rectal budesonide and systemic corticosteroids	Clinical: response<br>Biochemical: no response<br>Endoscopic: no response	Vedolizumab (3 infusions)	Clinical: remission<br>Biochemical: not assessable<br>Endoscopic: response

**Case 3**	Anti-PD-1/PD-L1	Clinical: grade 1 diarrhea<br>Biochemical: CRP 31 mg/L, calprotectin /<br>Endoscopic: Mayo 1<br>Anatomopathology: active/infectious type colitis	5-ASA	Clinical: response<br>Biochemical: not evaluated<br>Endoscopic: not evaluated	Systemic corticosteroids	Clinical: remission<br>Biochemical: remission<br>Endoscopic: no response	Vedolizumab (3 infusions)	Clinical: remission<br>Biochemical: remission<br>Endoscopic: remission
**Case 4**	Anti-PD-1/PD-L1	Clinical: grade 1 diarrhea<br>Biochemical: CRP 3.6 mg/L, calprotectin 329 μg/g<br>Endoscopic: Mayo 0<br>Anatomopathology: collagenous type colitis	Oral beclomethasone	Clinical: remission, after 2 months recurrence diarrhea<br>Biochemical: not assessable<br>Endoscopic: not evaluated	Oral budesonide	Clinical: response, on 3mg recurrence diarrhea<br>Biochemical: no response<br>Endoscopic: not assessable	Vedolizumab (5 infusions)	Clinical: remission<br>Biochemical: no response<br>Endoscopic: not assessable
**Case 5**	Anti-CTLA-4	Clinical: grade 2 diarrhea<br>Biochemical: CRP 7.1 mg/L, calprotectin /<br>Endoscopic: /<br>Anatomopathology: /	Systemic corticosteroids	Clinical: no response<br>Biochemical: remission<br>Endoscopic: not assessable<br>Anatomopathology: active/infectious type colitis	Methotrexate	Clinical: no response<br>Biochemical: not assessable<br>Endoscopic: no response	Vedolizumab (4 infusions)	Clinical: remission<br>Biochemical: not assessable<br>Endoscopic: remission
**Case 6**	Anti-CTLA-4 and anti-PD-1/PD-L1	Clinical: grade 3 diarrhea<br>Biochemical: CRP 78.8 mg/L, calprotectin 1122 μg/g<br>Endoscopic: Mayo 1	5-ASA, oral budesonide and systemic	Clinical: response, after 5 months of treatment, still	Vedolizumab (6 infusions)	Clinical: remission<br>Biochemical: response	/	/

		Anatomopathology: chronic/IBD-like type colitis	corticosteroids	diarrhea 5x/dayBiochemical: remission Endoscopic: remission		Endoscopic: not evaluated		
**Case 7**	Anti-CTLA-4 and anti-PD-1/PD-L1	Clinical: grade 2 diarrhea Biochemical: CRP 3.4 mg/L, calprotectin / Endoscopic: Mayo 2 Anatomopathology: active/infectious type colitis	Systemic corticosteroids	Clinical: no responseBiochemical: not assessable Endoscopic: response	Vedolizumab (3 infusions)	Clinical: remission Biochemical: remission Endoscopic: remission	/	/
**Case 8**	Anti-CTLA-4 and anti-PD-1/PD-L1	Clinical: grade 3 diarrhea Biochemical: CRP 13 mg/L, calprotectin / Endoscopic: Mayo 0 Anatomopathology: normal histopathological findings	Systemic corticosteroids	Clinical: remissionBiochemical: remission Endoscopic: no response	Vedolizumab (2 infusions)	Clinical: remission Biochemical: no response Endoscopic: remission	/	/
**Case 9**	Anti-PD-1/PD-L1	Clinical: grade 3 diarrhea Biochemical: CRP 55.9 mg/L, calprotectin 289 μg/g Endoscopic: Mayo 2 Anatomopathology: active/infectious type colitis, presence of apoptosis	Systemic corticosteroids	Clinical: no responseBiochemical: no response Endoscopic: no response	5 ASA, rectal budesonide and vedolizumab (4 infusions)	Clinical: response Biochemical: no response Endoscopic: response	Ustekinumab	Clinical: remissionBiochemical: remissionEndoscopic: not evaluated
**Case 10**	Anti-PD-1/PD-L1	Clinical: grade 2 diarrhea Biochemical: CRP 129.7 mg/L, calprotectin / Endoscopic: Mayo 1 Anatomopathology:	Systemic corticosteroids	Clinical: remission (after 2 months, recurrence of diarrhoea,	Vedolizumab (3 infusions)	Clinical: remission Biochemical: not evaluated	/	/

		chronic/IBD-like type colitis, presence of apoptosis		steroids were stopped since 4 weeks)Biochemical: response Endoscopic: not evaluated		Endoscopic: remission		
**Case 11**	Anti-CTLA-4 and anti-PD-1/PD-L1 and investigational drug (anti-IL8)	Clinical: grade 2 diarrhea Biochemical: CRP 10.4 mg/L, calprotectin 17 µg/g Endoscopic: Mayo 1 Anatomopathology: collagenous type colitis	Systemic corticosteroids	Clinical: initially remission, tapering corticosteroids with recurrence diarrhoeaBiochemical: no responseEndoscopic: no response	Vedolizumab (8 infusions)	Clinical: remission Biochemical: remission Endoscopic: remission	/	/

Table S7. Overview of patients with a relapse of ICI-induced colitis after rechallenge.

	**Type ICI**	**Evaluation at diagnosis**	**First line treatment**	**Evaluation response**	**Second line treatment**	**Evaluation response**	**Time to relapse colitis after rechallenge**	**Type ICI rechallenge**	**Evaluation**	**Treatment**	**Evaluation response**

**Case 1**	Anti-PD-1/PD-L1	Clinical: grade 1 diarrhea Biochemical: CRP 1.4 mg/L, calprotectin / Endoscopic: Mayo 2 Anatomopathology: active/infectious type colitis	5-ASA	Clinical: no response Biochemical: remission Endoscopic: no repsonse	Rectal budesonide (6mg) and beclomethasone (5mg)	Clinical: remission Biochemical: remission Endoscopic: response	44 weeks	Anti-PD-1/PD-L1	Clinical: RBPA, no diarrhea Biochemical: CRP 12.9 mg/L, calprotectin 1510 µg/g Endoscopic: Mayo 2 Anatomopathology: active/infectious type colitis	5-ASA, budesonide rectal	Clinical: remission Biochemical: remission Endoscopic: remission Anatomopathology: colonic mucosa with discrete non-specific changes
**Case 2**	Anti-PD-1/PD-L1 + anti-CTLA-4 + investigational drug (anti-IL8)	Clinical: grade 2 diarrhea Biochemical: CRP 10.4 mg/L, calprotectin 17 µg/g Endoscopic: Mayo 1 Anatomopathology: collagenou	Systemic corticosteroids	Clinical: remission Biochemical: no response Endoscopic: no	Vedolizumab (8 infusions)	Clinical: remission Biochemical: response Endoscopic: remission	8 weeks	Anti-PD-1/PD-L1 + TKI	Clinical: grade 3 diarrhea Biochemical: CRP 81 mg/L, calprotectin 1076 µg/g Endoscopic: Mayo 2 Anatomopathology:	Systemic corticosteroids	Not evaluated, oncological progression, †

		s type colitis		response					collagenous type colitis		
**Case 3**	Anti-PD-1/PD-L1 + anti-CTLA-4 + Carboplatin Paclitaxel	Clinical: grade 2 diarrhea Biochemical: CRP 5.7 mg/L, calprotectin / Endoscopic: Mayo 0 Anatomopathology: active/infectious type colitis	Beclomethasone	Clinical: remission Biochemical: not assessable Endoscopic: /	No second line treatment	No second line treatment	7 weeks	Anti-PD-1/PD-L1 + anti-CTLA-4	Clinical: grade 2 diarrhea Biochemical: CRP 49.7 mg/L, calprotectin 121 μg/g Endoscopic: Mayo 2 Anatomopathology: collagenous type colitis	Beclomethasone	Not evaluated, oncological progression, †
**Case 4**	Ant-PD-1/PD-L1 + anti-CTLA-4	Clinical: grade 3 diarrhea Biochemical: CRP 74.8 mg/L, calprotectin / Endoscopic	Systemic corticosteroids	Clinical: remission Biochemical: remission	No second line treatment	No second line treatment	11 weeks	Anti-PD-1/PD-L1	Clinical: grade 3 diarrhea Biochemical: CRP /, calprotectin / Endoscopic: Mayo 3	Budesonide (oral), systemic corticosteroids, vedolizumab (3 infusions)	Clinical: remission Biochemical: / Endoscopic: / Anatomopathology: / Oncological progression, †

		: Mayo 2 Anatomopathology: active/infectious type colitis		Endoscopic: /					Anatomopathology: chronic/IBD-like type colitis		

†: patient passed away.

Table S8. Infections leading to hospitalization.

Type of infection	Number of patients
Cholangitis	4
Bacteraemia	4
Infectious colitis	2
Urinary tract infection	2
Pneumonia	1
Diverticulitis	1
Liver abscess	1
Cellulitis	1
Total	16

Table S9. Causes of death.

**Mortality during follow-up?**	**% (*n*)<br>(total *n* = 77)**
No	45.5% (35)
Yes, due to oncological progression	39% (30)
Yes, due to colitis	0% (0)
Yes, due to other cause	3.9% (3)
Yes, cause unknown	11.7% (9)
**Cases with biologicals**	**Specific cause of death**
Infliximab case 1	Cause of death unknown
Infliximab case 2	Lost to follow-up
Infliximab case 3	Alive
Infliximab case 4	Alive
Infliximab case 5	Oncological progression
Infliximab case 6	Cause of death unknown

Infliximab case 7	Intracranial haemorrhage
Infliximab case 8	Oncological progression
Vedolizumab case 1	Alive
Vedolizumab case 2	Alive
Vedolizumab case 3	Alive
Vedolizumab case 4	Alive
Vedolizumab case 5	Alive
Vedolizumab case 6	Alive
Vedolizumab case 7	Oncological progression
Vedolizumab case 8	Alive
Vedolizumab case 9	Alive
Vedolizumab case 10	Alive
Vedolizumab case 11	Oncological progression
